# Perception of health risks in Lao market vendors

**DOI:** 10.1111/zph.12759

**Published:** 2020-08-19

**Authors:** Chanfong Philavong, Mathieu Pruvot, Daniel Reinharz, Mayfong Mayxay, Kongsy Khammavong, Phonesavanh Milavong, Sayapeth Rattanavong, Paul F. Horwood, Philippe Dussart, Bounlom Douangngeun, Watthana Theppangna, Amanda E. Fine, Matthew T. Robinson, Paul N. Newton

**Affiliations:** ^1^ Lao‐Oxford‐Mahosot Hospital Wellcome Trust Research Unit Microbiology Laboratory Mahosot Hospital Vientiane Lao PDR; ^2^ Ministry of Health Vientiane Lao PDR; ^3^ Institut de la Francophonie pour la Médecine Tropicale Vientiane Lao PDR; ^4^ Wildlife Conservation Society Health Program Bronx NY USA; ^5^ Département de Médecine Sociale et Préventive Université Laval Québec City QC Canada; ^6^ Centre for Tropical Medicine and Global Health Nuffield Department of Medicine University of Oxford Oxford UK; ^7^ Institute of Research and Education Development University of Health Sciences Vientiane Lao PDR; ^8^ Wildlife Conservation Society Lao PDR Program Vientiane Lao PDR; ^9^ College of Public Health, Medical and Veterinary Sciences James Cook University Townsville Australia; ^10^ Virology Unit Institut Pasteur du Cambodge Institut Pasteur International Network Phnom Penh Cambodia; ^11^ Department of Livestock and Fisheries National Animal Health Laboratory Ministry of Agriculture Vientiane Lao PDR

**Keywords:** bushmeat, food, health risk, Laos, markets, one health, risk perception, wildlife, zoonoses

## Abstract

Wet markets are a critical part of South‐East Asian culture and economy. However, their role in circulation and transmission of both endemic and emerging disease is a source of concern in a region considered a hotspot of disease emergence. In the Lao People's Democratic Republic (Lao PDR, Laos), live and dead wild animals are frequently found in wet markets, despite legislation against the bushmeat trade. This is generally considered to increase the risk of disease transmission and emergence, although whether or not wildlife vendors themselves have indeed increased incidence of zoonotic disease has rarely been assessed. In preparation for a future longitudinal study of market vendors investigating vendors’ exposure to zoonotic pathogens, we conducted a pilot survey of Lao market vendors of wildlife meat, livestock meat and vegetables, to identify demographic characteristics and potential control groups within markets. We also investigated baseline risk perception for infectious diseases among market vendors and assessed the association between risk perception and risk mitigation behaviours. The surveys conducted with 177 vendors revealed similar age, sex, ethnic background and geographical origin between vendor types, but differences in professional background and work history for livestock meat vendors. The perception of disease risk was very low across all vendors, as was the reported use of personal protective equipment, and the two appeared unrelated. Personal risk discounting and assumptions about transmission routes may explain this lack of association. This information will help inform the development of future research, risk communication and risk mitigation policy, especially in the light of the COVID‐19 pandemic.

**Impacts**

- Although wet markets are a critical part of South‐East Asian culture and economy, their role in circulation and transmission of infectious disease is poorly understood.
- In wet markets in the Lao PDR, the perception of disease risk was very low across all vendors, including wildlife vendors, as was the reported use of personal protective equipment, and the two appeared unrelated.
- There is a great need for public health educational engagement intervention of traders in wet markets to reduce the risk to traders and their wider communities.

## 1 INTRODUCTION

South‐East Asia is considered at high risk for emergence of infectious disease of wildlife origins (Jones et al., 2008; Morand, Jittapalapong, Suputtamongkol, Abdullah, & Huan, 2014). Over the last decade, epidemics of highly pathogenic avian influenza (HPAI) and severe acute respiratory syndrome (SARS) have led to increased interest in the role of markets as foci for disease emergence (Bell, Roberton, & Hunter, 2004; Brooks‐Moizer, Roberton, Edmunds, & Bell, 2009; Wang et al., 2006; Woo, Lau, & Yuen, 2006) and highlighted the need for One Health collaboration (Mwangi, de Figueiredo, & Criscitiello, 2016; Offeddu, Cowling, & Peiris, 2016). This has been further highlighted by the disastrous COVID‐19 pandemic.

Wet markets are a key component of the South‐East Asian economy and are a significant interface between wildlife, livestock and humans, as they bring live and dead animals of diverse species and origins in close proximity, potentially facilitating inter‐species transmission (Wang et al., 2006; Woo et al., 2006). At least part of the animal trade operates outside of official distribution chains and therefore bypasses slaughterhouses where inspection and testing for potential infectious agents would normally be carried out (Moy, 2001). The trade of wild animals and wildlife meat in wet markets is common in Laos (Bourgeois Luthi, Viravongsa Viravongsa, & Xaymounvong, 2012; Greatorex et al., 2016). There are ~ 400 villages and towns with permanent markets in Laos (www.decide.la) and Greatorex et al. (2016) documented at least 93 markets selling wildlife. In addition, it has been suggested that the inflow of wildlife of diverse origin, combined with the crowding of wet markets, and their strategic location in urban centres could increase the risk of pathogen spillover and further spread within the market, and in the diverse communities linked to these markets (Karesh & Noble, 2009; Swift, Hunter, Lees, & Bell, 2007). Wet markets represent small ‘mass gatherings’ of humans, livestock and wildlife risking enhanced transmission of both epidemic and endemic diseases. Endemic zoonotic pathogens that pose a transmission risk in markets in Laos include *Coxiella burnetti*, avian influenza virus, *Leptospira* spp., *Brucella* spp., rickettsia and diverse food‐borne bacteria and parasites (e.g. *Trichinella* spp.*, Taenia* spp.)(Burns et al., 2018; Holt et al., 2016; Mayxay et al., 2013, 2015).

There has been extensive research on bushmeat trade looking at the consequences on biodiversity loss (Fa, Peres, & Meeuwig, 2002; Milner‐Gulland & Bennett, 2003; Robinson & Bennett, 2004), their economic value (Kümpel, Milner‐Gulland, Cowlishaw, & Rowcliffe, 2010; Lescuyer & Nasi, 2016; Nielsen, Pouliot, Meilby, Smith‐Hall, & Angelsen, 2017), food security (Cawthorn & Hoffman, 2015; Golden, Fernald, Brashares, Rasolofoniaina, & Kremen, 2011) and disease risk (Karesh & Noble, 2009; Kilonzo, Stopka, & Chomel, 2013; Kurpiers, Schulte‐Herbrüggen, Ejotre, & Reeder, 2016; Pruvot et al., 2019; Saengthongpinit et al., 2019; Wolfe, Daszak, Kilpatrick, & Burke, 2005), with some controversy on the relative importance of these different components (Bonwitt et al., 2018; Pooley, Fa, & Nasi, 2015; Weber et al., 2015; Wilkie, 2006). However, evidence is still missing to allow objective weighing of the conservation, food security, livelihood and public health risks (Pruvot et al., 2019). For instance, there is still a limited understanding of the overall long‐term burden of disease attributable to bushmeat consumption in diverse rural and urban communities. In particular, the extent to which wildlife traders may be at increased risk of disease transmission from bushmeat has only been explored for a limited number of pathogens. In an investigation following the SARS outbreak, in Guangdong Province, China, the seroprevalence of SARS‐CoV IgG antibody in traders in live animal markets was 13% (none of whom were diagnosed with SARS), compared with 1%‐3% of those in three control groups (Bell et al., 2004; Centers for Disease Control and Prevention, 2003). Other research into the zoonotic disease risk for wildlife vendors has focused on the general identification of exposure along the commodity chains (Kamins et al., 2011; Paige et al., 2014), but much remains to be done to measure the public health risks of this occupation to inform appropriate interventions. Finally, although some information is available on the risk perception for infectious diseases among market traders in a few cultural contexts (Harrison et al., 2011; Kurscheid et al., 2015; LeBreton et al., 2006; Monroe & Willcox, 2006), there is still a limited understanding as to whether increased appropriate risk perception among vendors of unregulated meat, especially wildlife, will reduce risk‐taking behaviour (Monroe & Willcox, 2006).

In this study, we established baseline characteristics of market traders (demography, geographical origins) and their perception, behaviours and practices in regard to disease risk in Lao markets. In anticipation of further studies on the exposure of bushmeat vendors to pathogens, we compare bushmeat vendors to control groups among livestock meat and vegetable vendors. We also assessed the prevalence of risk‐reduction behaviours and their association with risk perception across vendor groups.

## 2 MATERIAL AND METHODS

A cross‐sectional study was performed with all consenting food sellers from three markets previously identified as hotspots for bushmeat trade (Greatorex et al., 2016) that sold all three food types (vegetables, domestic meat and wildlife meat). Markets were in Xieng Khouang Province in the north‐east (Phonsavan, 19°27′N 103°10′E), Bolikhamxai Province in central Laos (Lak Sao, 18°11' N 104° 58' E) and Salavanh Province in the south (Salavanh, 15°43′N 106°25′E). The target population consisted of all sellers of fresh produce who sold vegetables, domestic meat and/or wildlife meat. A questionnaire was collaboratively developed by a multi‐disciplinary team including veterinary epidemiologists, biologists, public health experts, infectious disease researchers and social scientists, and based on relevant literature (Becker & Maiman, 1975; Slovic & Peters, 2006; Triezenberg, Gore, Riley, & Lapinski, 2014). Prior to the main surveys, the questionnaire was tested and improved based on interviews conducted in a similar but distinct market. Data obtained during the questionnaire field testing were not included in this analysis.

All traders from each market were individually approached for interviews by a single interviewer who was a physician fluent in Lao language (CP). Vendors who gave informed written consent to participate in the study were interviewed using a questionnaire consisting of closed‐ended questions organized in four parts: (a) basic sociodemographic information; (b) perceived hazardousness of food sold in markets; (c) perceived benefits of preventive actions; and (d) sources of information. If vendors declined to be interviewed, they were asked if they would be able to briefly state the reason. Interviews took place in the market at a convenient place as judged by the vendors and were completed in ~15 min. Perceived risk was assessed on a five‐point scale (no = 0, little = 1, medium = 2, high = 3, or very high risk = 4). Additional direct observations on the use of protective equipment (boots, apron, gloves and masks) were conducted as vendors were approached for interviews. Data were collected from 18 April 2016 to 12 June 2016. Descriptive statistics were compiled on the three types of vendors (vegetable, domestic meat and wildlife meat) and compared using the appropriate parametric or non‐parametric test depending on the underlying data distribution using Stata v12 (College Station) and R‐3.5.1 (R Core Team, 2018). A *p* value of <.05 was considered statistically significant, with Bonferroni adjustment when needed. The association between perceived risk and vendor type or education level was assessed using a multinomial logistic regression. The survey questionnaire is available as supplementary File S1.

## 3 RESULTS

Of 187 vendors present in the markets and solicited to participate, 10 (5%) declined consent including 5 wildlife vendors because of fear of legal consequences and 5 livestock meat vendors who stated that they were too busy. Among the 177 respondents, 85 (48%) were vegetable vendors, 57 (32%) livestock meat vendors and 35 (20%) wildlife meat vendors. Five vendors who belonged to the Khamu, Katang, Ta‐Oy and Hmong ethnic groups required the assistance of a translator to Lao language to answer the questions. All but three of the participating vendors were women. Median (range) duration of formal education was 5 (0 to 15) years, with no significant differences in education levels between the three types of vendors (Kruskal–Wallis test, *p* = .12). The vegetable vendors had a slightly greater proportion of individuals reporting no education (24%), than livestock (12%) and wildlife meat (11%) vendors, but this difference was not statistically significant (chi‐square test, *p* = .13) (Table 1).

**Table 1:** Characteristics of the three types of vendors

Variables	Types of vendor		
	Vegetables	Livestock meat	Wildlife meat
	*N* = 85	*N* = 57	*N* = 35
Age (years), [mean (range)]	37.8 (14–64)	39.9 (22–64)	40.2 (25–65)
Distance from market to home (km) [median (range)]	2 (1–26)	2 (1–9)	2 (1–2)
Years working as vendor^a^ [median (range)]	2 (0.08–30)	10 (0.08–30)	1 (0.08–30)
Ethnic group			
Lao Loum	41 (48%)	37 (65%)	18 (51%)
Lao Theung and Lao Sung	44 (52%)	20 (35%)	17 (49%)
Education			
No education	20 (24%)	7 (12%)	4 (11%)
Primary school (year 1–6)	44 (52%)	31 (54%)	23 (66%)
Secondary school and above (>year 6)	21 (25%)	19 (33%)	8 (23%)
Trade as main occupation^a^	16 (19%)	4 (7%)	12 (34%)
If no, main occupation:			
Farmer^a^	13 (81%)	2 (50%)	9 (75%)
Trader	2 (13%)	1 (25%)	3 (25%)
Other	1 (6%)	1 (25%)	0

^a^ Indicates variables with statistically significant difference between vendor types (α = .05).

Wildlife vendors more often had another primary occupation (34%), than vegetable (19%) and livestock meat (7%) vendors (chi‐square test, *p* = .004), and overall farming was the main primary occupation. Occupations described prior to becoming a market vendor were similar and primarily farmer (63%), trader (14%) and housewife (10%), although livestock meat vendors had lower proportion of farmers (49%), compared with vegetable (67%) and wildlife meat (77%) vendors (chi‐square test, *p* = .015). There were also significant differences in the number of years spent working as a vendor between vendor types (Kruskal–Wallis test, *p* < .0001), with livestock meat vendors having worked longer in their jobs than wildlife meat and vegetable vendors (pairwise Wilcoxon rank sum test, Bonferroni‐adjusted *p* < .0001); wildlife and vegetable vendors had similar work durations (Bonferroni‐adjusted *p* = .22) (Table 1).

There were also no statistically significant differences between vendor types in their age distribution (ANOVA *F* = 0.923, *p* = .4) and distance from market to their homes (Kruskal–Wallis test, *p* = .4), with a median (range) of 2 (1–26) km, and 98% of vendors travelled 10 km or less. There was no difference between the vendors in the proportion of Lao loum (lowland Lao ethnic group) compared to other ethnic groups (chi‐square test, *p* = .14), although the livestock meat vendors had a slightly lower ethnic diversity (*n* = 7 ethnic groups) compared to the vegetable (*n* = 13) and wildlife meat (*n* = 9) vendors.

All wildlife meat vendors sold both live and dead wild animals, while the domestic meat sellers only sold slaughtered livestock (although live domestic animals were sold elsewhere in the market). Most vegetable vendors sourced their vegetables from other vendors (76%), while the rest farmed the vegetables themselves. Livestock meat was primarily obtained from slaughterhouses (70%), but some vendors reported slaughtering the animal themselves (19%) or obtaining the meat from other vendors/retailers (11%). Wildlife meat vendors rarely hunted the bushmeat themselves (6%), and most often bought it from a hunter (49%) or another vendor/middleman (46%).

The type of installations (table/stall vs. sitting on a mat on the market floor) used by vendor types significantly differed; the proportion of vendors using table/stall structures was highest for livestock meat (100%) vendors, 61% for vegetable vendors, and 40% for wildlife meat vendors (chi‐square test, *p* < 0001). The median duration of education was 1 year lower for vendors using mats than vendors using tables/stalls (Wilcoxon rank sum test, *p* = .01). For vendors on mats, this activity was less often a primary occupation (69%) than for stall vendors (88%, chi‐square test, *p* = .004), and a more recent occupation (median time as a vendor was 5 years for table vendors and 0.75 years for mat vendors; Wilcoxon rank sum test, *p* < .0001). Vendors on mats tended to live further away from the market (Wilcoxon rank sum test, *p* = .05), although this was mainly as a consequence of the high proportion of wildlife vendors using mats. However, there was no significant difference in vendor age (ANOVA *F* = 2.0, *p* = .16) and frequency of different ethnicities using mats (chi‐square test, *p* = .12).

**Figure 1:**
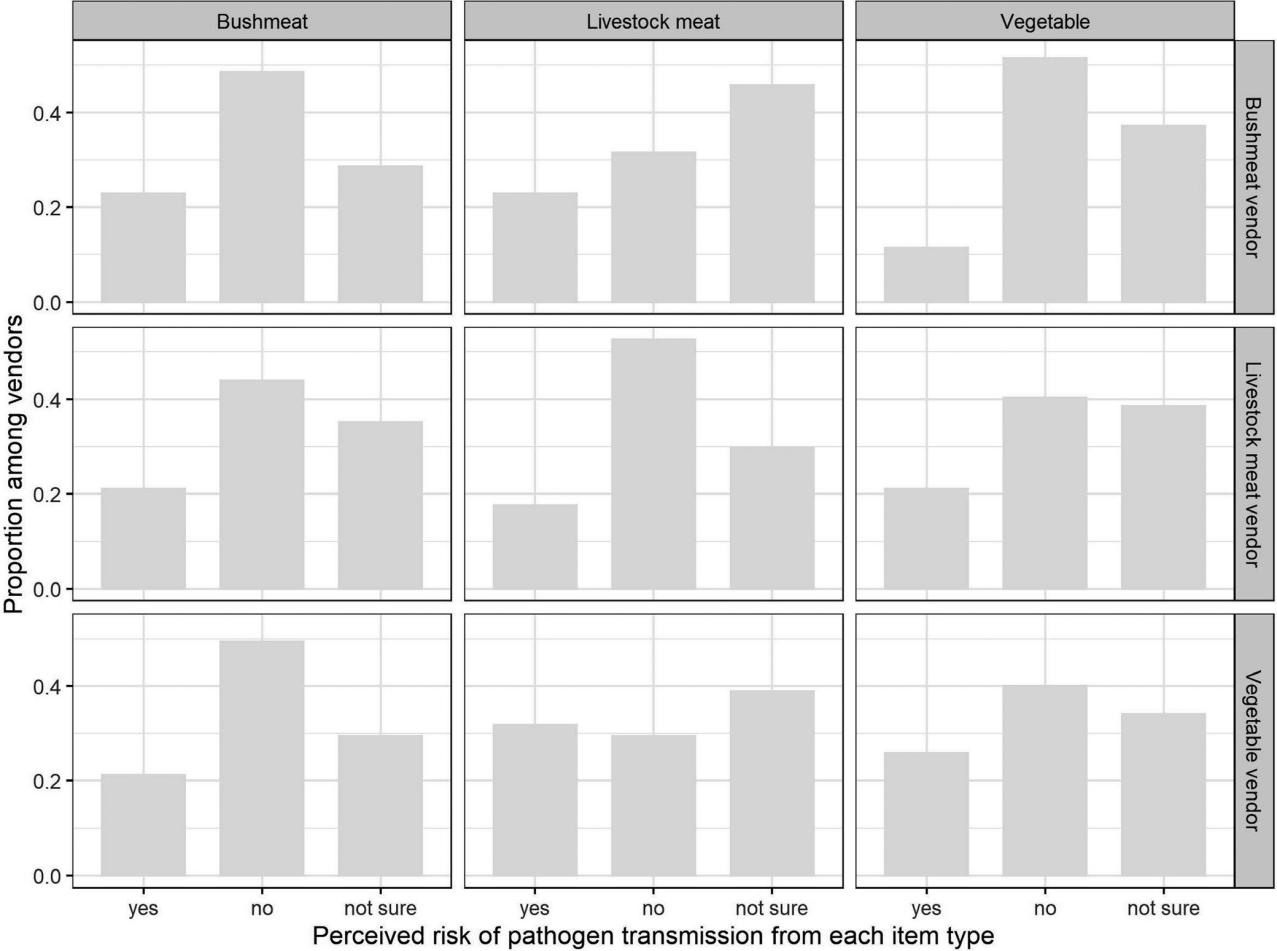
Perception of the disease transmission risk for each vendor type (rows) and each product type (columns). Each panel indicates the proportion of respondents who selected one of the three possible answers (“yes,” ‘no” and “not sure”)

Among vegetable, livestock meat and wildlife meat vendors, 26%, 17% and 23%, respectively, stated that their food products could transmit disease to humans. Remarkably, the proportion of respondents who indicated they were “not sure” was consistently around 30% for all vendors. When comparing the response of the different vendor types for the different food items, vegetable and bushmeat vendors reported more often that there was “no risk” of disease transmission for their products, while the livestock meat vendors reported more frequently that livestock meat was not transmitting diseases (chi‐square test, *p* = .014) (Figure 1).

The diseases and syndromes cited by vendors as transmitted from vegetables were abdominal pain (7 respondents), diarrhoea (9), common cold (3), parasites (2), allergy (1) and hypertension (1); from livestock meat: diarrhoea (4), abdominal pain (2), parasites (2) and common cold (2); and from bushmeat: abdominal pain (2), malaria (2) and parasites (2). Most vendors considered that the more likely transmission route was through consumption (79%), followed by direct contact (19%), with no statistically significant differences between vendor types (chi‐square test, *p* = .4). For vegetable vendors who reported potential transmission, their assessment of risk on a 5‐point scale was significantly lower when referring to the product sold on their stall than for the product in general (paired Wilcoxon rank test, *p* = .019); this was not observed for livestock and wildlife meat vendors.

**Figure 2:**
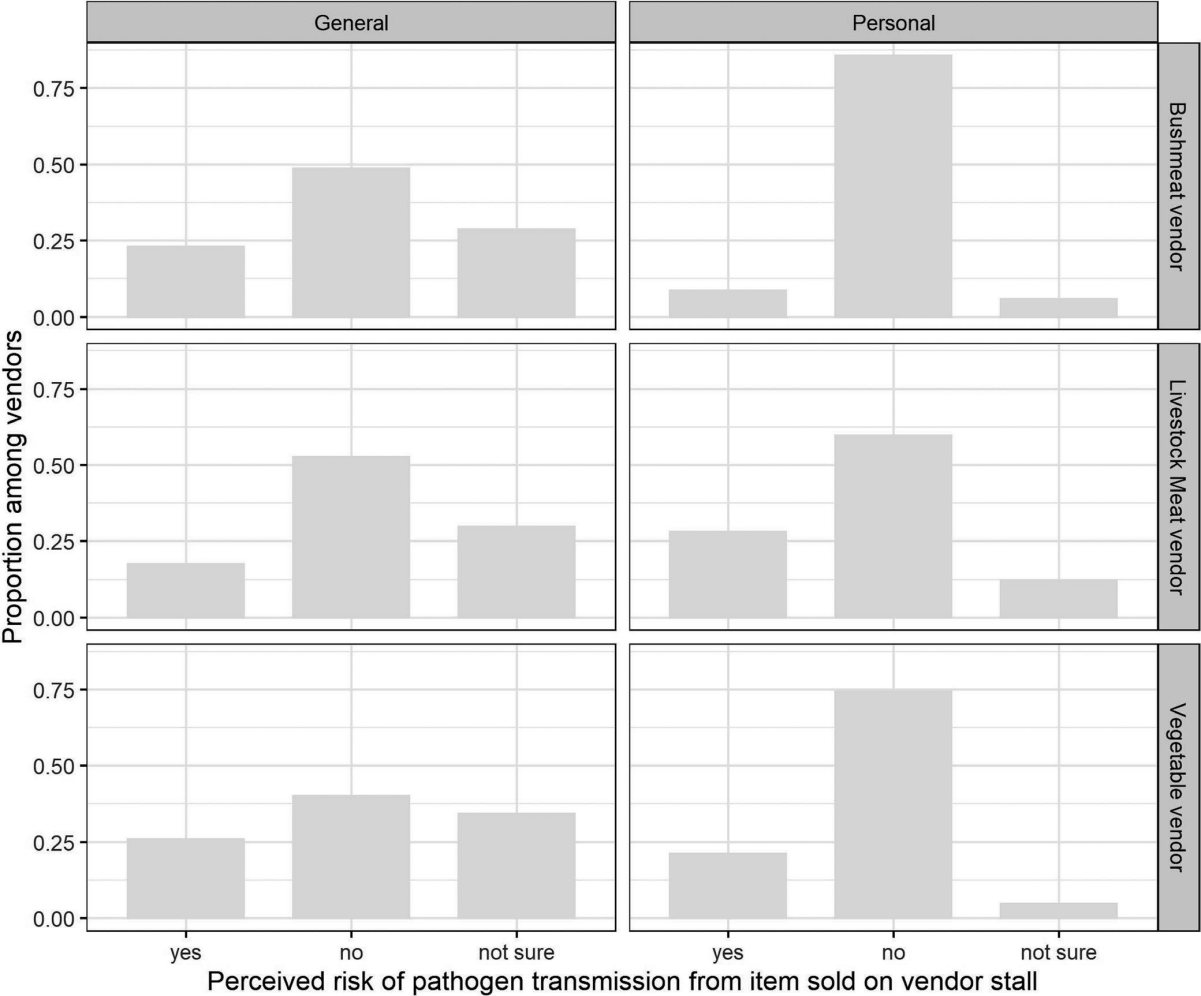
Risk perception for general and personal risk of disease transmission from traded products among market vendor types

When asked about the health risks related to their activity as vendors, most (72%) considered that their job did not put their health at risk. This was particularly clear among the wildlife vendors, of whom 86% did not consider their health at risk from selling bushmeat, and were found to have lower odds of responding “yes” regarding the existence of a personal risk than other vendors in a multinomial logistic regression (OR = 0.21; 95% CI: 0.05–0.80). In addition, the proportion of vendors who reported that they had “no risk” was higher when asked about their personal risk compared to when they were asked about risk in general, and this was consistent for vegetable vendors (chi‐square test, *p* < .001), livestock meat vendors (chi‐square test, *p* = .055) and bushmeat vendors (chi‐square test, *p* = .0037) (Figure 2). This low perceived risk was mainly attributed to the belief that they sold healthy food. Vegetable vendors often linked this belief to the idea that their products were “organic,” “healthy” and “natural.” Livestock meat vendors mentioned that the meat was healthy because it was often provided by slaughterhouses where veterinary control was done. For some bushmeat vendors (13 respondents), not being involved in the hunting and killing of the animal seemed to be perceived as reducing their risk. When vegetable and livestock meat vendors did perceive a risk, it was most often associated with the potential presence of chemicals in the food. The odds of perceiving a personal risk increased with the number of years of education (OR = 1.16; 95% CI: 1.04–1.31 per year).

Consistently across vendor types, masks and gloves had very low levels of reported usage by vendors (Table 2). Boots were reported to be used similarly across vendor types (chi‐square test, *p* = .28). The reported use of aprons varied between vendor types (chi‐square test, *p* = .0004) and was particularly frequent in livestock meat vendors. When aprons were used, it was mainly out of concern for vendor cleanliness. There was discordance between the reported and observed use of masks, gloves and boots, although consistency was greater for aprons. Overall, there was no association between the perceived risk of disease transmission and the reported use of protective equipment.

**Table 2:** Reported and observed use of personal protective equipment by vendors

Protective equipment	Vegetables		Livestock meat		Wildlife meat	
	*N* = 85	%	*N* = 57	%	*N* = 35	%
Mask—reported	17	20	10	18	5	14
Mask—observed	2	2	1	2	0	0
Gloves—reported	11	13	10	18	6	17
Gloves—observed	2	2	2	4	1	3
Apron—reported	55	65	51	89	19	54
Apron—observed	34	40	51	89	11	31
Boots—reported	30	35	13	23	11	31
Boots—observed	4	5	1	2	0	0

Finally, among vegetable, livestock meat and wildlife meat vendors, 5%, 14% and 9%, respectively, indicated they received external information about disease potentially transmitted by their product (chi‐square test, *p* = .15). Sources cited included television (*n* = 7), radio (*n* = 4), health (*n* = 3) professional and newspapers (*n* = 1).

## 4 DISCUSSION

This study was conducted to obtain key demographic information on different vendor types in order to facilitate the planning of future studies on zoonotic disease exposure among Lao vegetable, livestock meat and wildlife meat vendors, and to compare perception of the health risk related to selling these products.

Although we selected markets to be representative of both typical wet market and wildlife trade hotspots, and that we were able to interview most vendors on these three markets, extrapolation to the population of market vendors across Lao PDR should be made very cautiously, as the limited number of markets may limit representativeness, and our overall sample size was low (resulting in low statistical power). Vendors interviewed in these three markets were mainly women with a wide range of educational levels. Age, geographical origin and ethnic background between vendor types were comparable. The main differences between vendor types were with their professional background and work history. Livestock meat vendors reported more frequently that this was their primary occupation, which they had practiced for a longer time, and they came less frequently from a farming background as compared to the other vendor categories. This may indicate that livestock meat vendors involve themselves in this activity as a professional career compared to wildlife and vegetable vendors who may more often choose this activity as an opportunistic and secondary occupation. Hence, wildlife and vegetable vendors were more comparable, which may influence future prospective study design. An important consideration for study design is the apparent differences observed between vendors based on their use of stalls or floor mats. Floor mat vendors had a shorter history as vendors, had less formal education and travelled further distances to the market. Given the different proportions of floor mat vendors between vendor types, part of the difference between vendor types may be attributed to this confounding factor. Although not investigated in this study, it is likely that market rent for floor mats is less than for tables/stalls and this aspect should be investigated in future studies.

It is noteworthy that most vendors, regardless of vendor types, came from within a distance of 10 km from the market. In a context of assessing the risk of zoonotic disease exposure, this is important information on the potential disease spread around markets. This does not account for market customers who may come from much greater distances (unpublished data). Wildlife meat vendors sold both dead and live animals, the latter probably representing a greater risk of disease exposure. Livestock meat vendors did not handle live animals at the market, but 19% reported slaughtering animals themselves outside of slaughterhouses. The extent to which different exposures among vendor types result in different incidence of zoonotic disease is a question of great interest that remains to be addressed. In particular, comparing the risks of zoonotic disease transmission between bushmeat and livestock meat vendors is of great relevance to risk communication and policy development.

The study shows that regardless of the type of food item sold, market vendors perceived low health risks. Vendors perceived a lower risk for the type of item they sell and arguably are more familiar with. Similarly, vendors had a lower personal risk perception than general risk perception. This was also apparent among vegetable vendors who systematically ranked the risk level lower when referring to the vegetables they sell rather than vegetables in general. This type of risk discounting related to familiarity, knowledge and preference is common in the field of public health risk prevention (Monroe & Willcox, 2006; Ortendahl, 2007; Stringer et al., 2004).

The main transmission route perceived by vendors to be involved in pathogen transmission was consumption. This may partly explain why vendors discounted their risk of contracting disease from their product, as vendors would be more involved with handling the product than consuming it. When a risk was perceived, it was also frequently associated with the use of pesticides and chemicals, which again would be more relevant to consumers than vendors.

The use of protective equipment was low across vendor types, except for the higher use of aprons in livestock meat vendors. This may reflect the overall low‐risk perception for disease transmitted by traded products. There is also no legal obligation to use personal protective equipment in Lao markets, as is the case in many other developing countries (Havelaar et al., 2013). Moreover, there was no association between the risk perception and the use of protective equipment. This lack of association between risk perception and risk avoidance behaviours may be related to similar personal risk discounting as mentioned earlier, and assumptions made by vendors on the main transmission route involved, resulting in protective equipment being perceived as irrelevant.

The overall low level of risk perception and the frequency of vendors reporting being “not sure,” and the association with the years of education pointed out to the opportunity for community health engagement and education. Understanding current risk perception and how risk perception translates into risk mitigation behaviours is critical to the appropriate targeting of community health engagement. These observations suggest that increasing awareness about common endemic infectious diseases would be necessary, especially regarding the details of how these infections are transmitted. The importance of this has escalated considerably with the likely origin of the COVID‐19 pandemic in the wildlife trade.

Further prospective assessments of how community engagement could positively influence risk perception and risk avoidance behaviours will be important to assess the effectiveness of health engagement campaigns as a prevention strategy.

## CONFLICT OF INTEREST

The authors declare that they have no conflicts of interest in relation to this paper.

## ETHICAL STATEMENT

This study was approved by the Lao National Institute of Public Health's National Ethics Committee for Health Research (certificate #020) and by the directors of the three markets. The study conforms to the Declaration of Helsinki.

## Supporting information

Supplementary Material
