# Supplementary material for: Perception of health risks in Lao market vendors
Source: Zoonoses Public Health. 2020 Aug 19;67(7):796–804. doi: 10.1111/zph.12759 (PMC7461205; doi:10.1111/zph.12759)
Supplement: Supplementary file 1 — Supplementary Material [file ZPH-67-796-s001.pdf]

Supplementary Material – Questionnaire for 'Perceptions of health risks in Lao market vendors' by Philavong *et al.*

NB. The questionnaire was used in Lao and has been translated from Lao to English

1. Questionnaire for vegetable sellers

Form ID: _ _ _; Date _____ /_____ / ______

I. Information on markets and vendors

N°	Questions	Answer
1.	Province name	1. Xiengkhoung<br>2. Vientiane capital<br>3. Salavanh
2.	Market name	1. Phonsavanh<br>2. Dongmakkhaiy<br>3. Salavanh
3.	Age	…………. Years
4.	Gender	1. Male<br>2. Female
5.	Marital status	1. Single<br>2. Married<br>3. Divorced<br>4. Widowed<br>5. Separated
6.	Ethnicity	……………
7.	Schooling years	……… years
8.	Duration of trading years	………..years
9.	Distance from vendor's house to market	…….. km
10.	Previous job	1. Farmer<br>2. Worker<br>3. Official<br>4. Other…………………
11.	Residence (village or phone number)	……………….. Tel. ………… ….

II. Perceived hazardousness of food sold in markets

12.	What diseases do you think vegetable sellers could be at risk of ?	1. Diarrhea<br>2. Parasite<br>3. Food poisoning<br>4. Virus (hepatitis A)<br>5. Other ………………..
13.	How are the diseases transmitted?	1. Contact<br>2. Eating

		3. Not washing hands after selling vegetable<br>4. Other …………………
14.	Do you think the diseases are severe?	1. Yes<br>2. No<br>3. Do not know
15.	Are you afraid about infection from vegetables you sell ?	1. A lot<br>2. Little<br>3. Never
16.	Are you aware of any diseases been transmitted from selling vegetables in past year ?	1. Yes<br>2. No<br>3. Do not know

III. Perceived benefits of preventive action

17.	Disease transmission from selling vegetables can be prevented by these equipment ?	1. Put on mask<br>2. Gloves<br>3. Wear a protective suit<br>4. Wear boots<br>5. Wash hands after work<br>6. Other………………….
18.	Disease transmission from selling vegetables can be prevented by vaccination ?	1. Yes<br>2. No<br>3. Do not know
19.	Barriers for prevention are:	1. Uncomfortable<br>2. Equipment is expensive<br>3. Rarely available<br>4. Other…………..
20.	Barriers to vaccine are:	1. Expensive<br>2. Far from hospital<br>3. Do not know<br>4. Other

IV. Likelihood of vegetable sellers to undertake preventive health action to reduce risk

21.	If you know protective equipment and vaccine can protect you from disease, do you use it ?	1. Yes<br>2. No
22.	If yes, how often do you use it?	1. Always<br>2. Sometimes<br>3. Never

V. Information sources

23.	How do you know about information related to health risks in markets ?	1. Television<br>2. Newspapers<br>3. Radio<br>4. Health professionals<br>5. Other ……………….

2: Questionnaire for livestock meat sellers

Form ID: _ _ _; Date ____ /____ / ____

I. Information on markets and vendors

N°	Questions	Answer
1.	Province name	1. Xiengkhoung<br>2. Vientiane capital<br>3. Salavanh
2.	Market name	1. Phonsavanh<br>2. Dongmakkhaiy<br>3. Salavanh
3.	Age	…………. years
4.	Gender	1. Male<br>2. Female
5.	Marital status	1. Single<br>2. Married<br>3. Divorced<br>4. Widowed<br>5. Separated
6.	Ethnicity	……………
7.	Schooling years	……… years
8.	Livestock meat sold	1. Beef<br>2. Buffalo<br>3. Pork<br>4. Chicken<br>5. Duct<br>6. Fish<br>7. Other …………….
9.	During of trading years	………….years
10.	Distance from vendor's house to market	…….. km
11.	Previous job	1. Farmer<br>2. Worker<br>3. Official<br>4. Other
12.	Residence (village or phone number)	……………….. Tel: …………..

II. Perceived hazardousness of food sold in market

13.	What diseases do you think livestock meat sellers could be at risk of ?	1. Parasites<br>*2. Streptococcus suis*<br>3. H5N1<br>4. Diarrhea<br>5. Virus (hepatitis E)

		6. Other……………..
14.	How are the diseases transmitted?	1. Contact<br>2. Eat<br>3. Not washing hand after selling meat<br>4. Other………………
15.	Do you think the diseases are severe?	1. Yes<br>2. No<br>3. Do not know
16.	Are you afraid about infection from selling livestock meat ?	1. A lot<br>2. Little<br>3. Never
17.	Are you aware of disease transmitted from livestock meat in past year?	1. Yes<br>2. No<br>3. Do not know

III. Perceived benefits of preventive action

18.	Disease transmission from selling livestock meat can be prevented by these equipment ?	1. Put on mask<br>2. Gloves<br>3. Wear a protective suit<br>4. Wear boots<br>5. Wash hand after work<br>6. Other…………..
19.	Disease transmission from selling livestock meat can be prevented by vaccination ?	1. Yes<br>2. No<br>3. Do not know
20.	Barriers to prevention are:	1. Uncomfortable<br>2. Equipment is expensive<br>3. Rare<br>4. Other………………
21.	Barriers to vaccine are:	1. Expensive<br>2. Far from hospital<br>3. Do not know<br>4. Other………………..

IV. Likelihood of livestock meat sellers to undertake some preventive health action to reduce the risk

22.	If you know protective equipment and vaccine can protect you from disease, will you use or not?	1. Yes<br>2. No
23.	If yes, how often do you use it?	1. Always<br>2. Sometimes<br>3. Never

V. Information sources

24.	How do you know about information related to health risks in markets ?	1. Television<br>2. Newspapers<br>3. Radio<br>4. Health professional<br>5. Other ……………..

3: Questionnaire for wildlife sellers

Form ID: _ _ _; Date ____ /____ / ____

I. Information on market and vendors

N°	Questions	Answer
1.	Province name	1. Xiengkhoung<br>2. Vientiane capital<br>3. Salavanh
2.	Market name	1. Phonsavanh<br>2. Dongmakkhaiy<br>3. Salavanh
3.	Age	…………. years
4.	Gender	1. Male<br>2. Female
5.	Marital status	1. Single<br>2. Married<br>3. Divorced<br>4. Widowed<br>5. Separated
6.	Ethnicity	……………
7.	Schooling years	……… years
8.	Type of wildlife sold	1. Birds<br>2. Mammals<br>3. Reptiles & amphibians
9.	If you obtain wildlife alive do you kill them before selling or do you sell them without killing	1. Kill<br>2. Not kill
10.	How long do you keep wildlife before selling?	……….…days
11.	How long do you keep dead wildlife before selling it?	…………days
12.	If you sell wildlife meat in parts what do you do with the wildlife meat remaining?	1. Throw away<br>2. Eat<br>3. Sell<br>4. Other ……………….
13.	Duration of trading years	………….years
14.	Distance from vendor's house to market	…….. km
15.	Previous job	1. Farmer<br>2. Worker<br>3. Official<br>4. Other
16.	Residence (village or phone number)	……………….. Tel: …………..

II. Perceived hazardousness of food sold in market

17.	What diseases do you think wildlife sellers could be at risk of ?	1. Parasites<br>2. Leptospirosis<br>3. Rickettsia<br>*4. Streptococcus suis*<br>5. H5N1<br>6. Diarrhea<br>7. Other……………
18.	How are these diseases transmitted?	1. Contact<br>2. Eating<br>3. Not washing hands after selling wildlife<br>4. Other…………….
19.	Do you think the diseases are severe?	1. Yes<br>2. No<br>3. Do not know
20.	Are you afraid about infection from selling wildlife ?	1. A lot<br>2. Little<br>3. Never
21.	Are you aware of any diseases been transmitted from selling wildlife in past year ?	1. Yes<br>2. No<br>3. Do not know

III. Perceived benefits of preventive action

22.	Disease transmission from selling wildlife can be prevented by these equipment :	1. Put on mask<br>2. Gloves<br>3. Wear protective suit<br>4. Wear boots<br>5. Wash hands after work<br>6. Other ……………..
23.	Disease transmission from selling wildlife can be prevented by vaccination	1. Yes<br>2. No<br>3. Do not know
24.	Barriers for prevention are:	1. Uncomfortable<br>2. Equipment is expensive<br>3. Rarely available<br>4. Other ……………..
25.	Barriers to vaccine are:	1. Expensive<br>2. Far from hospital<br>3. Do not know<br>4. Other…………………

IV. Likelihood of the wildlife sellers to undertake preventive health action to reduce the risk

26.	If you know protective equipment and vaccine can protect you from disease, do you use it ?	1. Yes<br>2. No
27.	If yes, how often do you use it?	1. Always<br>2. Sometimes<br>3. Never

V. Information sources

28.	How do you know about information related to health risks in markets ?	1. Television<br>2. Newspapers<br>3. Radio<br>4. Health professional<br>5. Other………………..
